# Powder 3D Printing of Bone Scaffolds with Uniform and Gradient Pore Sizes Using Cuttlebone-Derived Calcium Phosphate and Glass-Ceramic

**DOI:** 10.3390/ma15155139

**Published:** 2022-07-24

**Authors:** Francesca Cestari, Yuejiao Yang, Janka Wilbig, Jens Günster, Antonella Motta, Vincenzo M. Sglavo

**Affiliations:** 1 Department of Industrial Engineering, University of Trento, Via Sommarive 9, 38123 Trento, Italy; yuejiao.yang@unitn.it (Y.Y.); antonella.motta@unitn.it (A.M.); vincenzo.sglavo@unitn.it (V.M.S.); 2 BIOtech Research Center, University of Trento, Via delle Regole 101, 38123 Trento, Italy; 3 Division of Advanced Multi-Materials Processing, Bundesanstalt für Materialforschung und -Prüfung BAM, Unter den Eichen 44-46, 12203 Berlin, Germany; janka.wilbig@bam.de (J.W.); jens.guenster@bam.de (J.G.)

**Keywords:** cuttlefish, biphasic calcium phosphate, binder jetting, scaffold geometry, Hausner ratio, bioactivity

## Abstract

The pore geometry of bone scaffolds has a major impact on their cellular response; for this reason, 3D printing is an attractive technology for bone tissue engineering, as it allows for the full control and design of the porosity. Calcium phosphate materials synthesized from natural sources have recently attracted a certain interest because of their similarity to natural bone, and they were found to show better bioactivity than synthetic compounds. Nevertheless, these materials are very challenging to be processed by 3D printing due to technological issues related to their nanometric size. In this work, bone scaffolds with different pore geometries, with a uniform size or with a size gradient, were fabricated by binder jetting 3D printing using a biphasic calcium phosphate (BCP) nanopowder derived from cuttlebones. To do so, the nanopowder was mixed with a glass-ceramic powder with a larger particle size (45–100 µm) in 1:10 weight proportions. Pure AP40mod scaffolds were also printed. The sintered scaffolds were shown to be composed mainly by hydroxyapatite (HA) and wollastonite, with the amount of HA being larger when the nanopowder was added because BCP transforms into HA during sintering at 1150 °C. The addition of bio-derived powder increases the porosity from 60% to 70%, with this indicating that the nanoparticles slow down the glass-ceramic densification. Human mesenchymal stem cells were seeded on the scaffolds to test the bioactivity in vitro. The cells’ number and metabolic activity were analyzed after 3, 5 and 10 days of culturing. The cellular behavior was found to be very similar for samples with different pore geometries and compositions. However, while the cell number was constantly increasing, the metabolic activity on the scaffolds with gradient pores and cuttlebone-derived powder decreased over time, which might be a sign of cell differentiation. Generally, all scaffolds promoted fast cell adhesion and proliferation, which were found to penetrate and colonize the 3D porous structure.

## 1. Introduction

When designing a scaffold for bone tissue engineering (BTE), one of the most important features to consider is porosity. In natural human bones, porosity allows for the ramification of blood vessels, stores and protects bone marrow, hosts the bone cells (osteocytes) in holes, called lacunae, and connects them through small channels, called canaliculi [1,2]. Porosity also directly affects bones’ mechanical properties, and it is considered by some authors [3] to be the only difference between cancellous bone (spongy, ~50–90% porosity [4]) and cortical bone (compact, ~5–10% porosity [4]). The geometry of the scaffold porosity was found to have a significant influence on the cellular response, as tissue regeneration increases with curvature and is favored by concave surfaces [5]. Pore size and distribution can be also important, since natural bone possesses a gradient in its porous structure from cortical to cancellous. Nevertheless, it is still not clear if a gradient pores scaffold is more effective than one with uniform pore distribution [6]. It is therefore obvious that the full control of the scaffold’s porosity (size, shape, orientation, size distribution, interconnection, etc.) is highly desirable; for this reason, 3D printing represents a very attractive technology for BTE.

By using a three-dimensional Computer Aided Design (CAD) model, additive manufacturing (AM) technologies allow for fully designing the scaffold’s pores and open up the possibilities for customizing scaffolds based on medical imaging [7,8]. Polymers, metals, ceramics and composite materials can all be 3D printed with different AM processes, such as FDM (fused deposition modelling), SLA (stereolithography), SLS (selective laser sintering), SLM (selective laser melting), robocasting or DIW (direct ink writing), and binder jetting or P-3DP (powder 3D printing) [9,10]. So far, AM technologies have been more successful for metals and polymers, due to the fact that ceramic materials are not easy to print because of their challenging process requirements, such as sintering [11]. Indeed, even a small defect can grow upon the heat treatment, compromising the final properties of the material [12]. Nevertheless, bioceramics, and especially calcium orthophosphates (CaPs), are particularly interesting for BTE because of their chemical similarity to the mineral phase of natural bone. These materials are non-toxic, biocompatible and also osteoconductive (able to support bone tissue ingrowth), promoting osteoblasts’ adhesion and proliferation [13,14].

Many CaPs, such as hydroxyapatite (HA), β-tricalcium phosphate (β-TCP), α-TCP, biphasic HA/β-TCP, tetracalcium phosphate and octacalcium phosphate, have been used to produce scaffolds by SLS, SLA, P-3DP and DIW [15,16]. In the SLS process, the ceramic powders are usually blended with polymers such as PLA [17], PDLLA [18], PCL and PEEK [19] to form composite materials, in order to avoid the high operating temperatures that are required for sintering [10]. In P-3DP, SLA and DIW, instead, powders are used alone or in suspensions or pastes, with this allowing for the fabrication of pure CaPs scaffolds. This is because the particles are bound together by using liquid binders, photocurable polymers or organic substances, which are subsequently eliminated during the sintering phase [20].

It has been shown that the bioactive properties of CaPs can be further improved by doping them with beneficial elements that are naturally present in the bone apatite, such as zinc, strontium, silicon, manganese and magnesium [21,22]. These elements can stimulate bone formation (Zn, Mg, Sr, B) and promote osteoblastic adhesion (Mg, Zn) [23], proliferation and differentiation (Si, Zn, Cu) [24]. The incorporation of said elements can be easily achieved by extracting CaPs from natural resources such as corals, eggshells, mussel shells and cuttlebones, with processes that usually result in nanometric, carbonated and non-stoichiometric HA, which is more similar to the bone mineral phase than pure stoichiometric HA [25,26].

Ion-doped and bio-derived CaPs are therefore very interesting for BTE and, consequently, for additive manufacturing, but to the authors’ knowledge, only a few studies about the 3D printing of these materials have been reported. Two recent research works reported the fabrication of Sr-doped nano-HA scaffolds by DIW, showing better cell proliferation and differentiation in vitro for the samples with 5% [27] and 10–15% Sr [28]. Recently, Mocioiu et al. [29] used nano-HA derived from *Rapana venosa* shells to 3D print bone scaffolds by DIW, finding that the cell viability was similar to that of synthetic HA. Cuttlefish bone powder was instead used in another work [30] by mixing it with PLLA to fabricate composite scaffolds via the SLS process. The hydrothermal transformation to HA was performed after printing, and the scaffolds were proven to be non-cytotoxic. In a previous work [31], we also fabricated composite scaffolds by FDM using PCL and nano-HA derived from cuttlefish bones, eggshells and mussel shells, finding that bio-derived HAs promote good cell adhesion and proliferation.

However, no study has reported the use of P-3DP to produce ion-doped or bio-derived CaPs, very likely because these powders are usually nanosized, while the P-3DP process requires larger particle sizes or granules to achieve good powder flowability and densification [11]. Nevertheless, P-3DP is an attractive process when compared to other AM technologies, with it having the advantages of working with the widest selection of materials, not inducing thermal stresses and distortions, and producing large volumes of parts at relatively low cost [32]. Another advantage for biological applications is the residual porosity in the struts, which is related to the packing density of the powder bed, and which can improve the scaffold’s bioactivity [11].

In the present work, we produced porous scaffolds characterized by uniform pores or with gradient pore sizes via P-3DP. Nano-CaP powder derived from cuttlefish bones was used, overcoming the technological issue related to the fine particle size by mixing it with a glass-ceramic powder with larger particle sizes (45–100 µm). The maximum amount of nano-CaP that it was possible to incorporate while obtaining a good printing quality was 10 wt%. A comparative analysis between scaffolds with uniform or gradient pores and with or without cuttlebone-derived CaP was carried out by studying cell adhesion, proliferation and metabolic activity in vitro using human mesenchymal stem cell (hMSCs) lines.

## 2. Materials and Methods

### 2.1. Materials

Cuttlefish (*Sepia officinalis*) bones were collected locally in a fish shop, rinsed under tapped water, boiled in demineralized water for 10 min and dried at 100 °C for 24 h. Then, they were ground into powder by a centrifugal mill (Retsch S100, Haan, Germany) and sieved through a 300 µm mesh to eliminate the larger particles. The transformation into CaP was performed by ball-milling the cuttlebone powder in a ~3.4 vol% H_3_PO_4_ aqueous solution, using stoichiometric proportions (Ca/P = 1.67; 91.6% CaCO_3_ in the cuttlebone powder, as estimated by preliminary thermo-gravimetric analysis). The phosphoric acid, ~85% H_3_PO_4_, CAS: 7664-38-2, was purchased from CARLO ERBA Reagents (Emmendingen, Germany). Ball-milling was carried out for 30 min in a Turbula^®^ (WAB-Group, Muttenz, Switzerland) mixer at 101 rpm by using a 250 mL polyethylene bottle (vial filling ~25%) with zirconia balls (ball mass = 0.5 g; balls-to-powder mass ratio = 10:1). After milling, the slurry was dried overnight in an oven at 150 °C, and the obtained powder was heat treated at 800 °C (heating rate 10 °C/min, dwell 5 h, free cooling in the furnace) in order to eliminate all the organics. This was done after the first printing trials because the organic part was shown to deteriorate during the printing heating phase, leading to the failure of the printing process.

The glass-ceramic material, named AP40mod, had been previously produced at BAM (Berlin, Germany). It is a glass-ceramic with a composition of (wt%) 39.60 SiO_2_, 32.04 CaO, 11.26 P_2_O_5_, 2.01 Na_2_O, 3.52 MgO, 5.03 CaF_2_, and 6.54 TiO_2_, and its preparation is described in [33]. The sieved fraction between 45 µm and 100 µm was used for the printing process as produced or mixed with 10 wt% of cuttlebone-derived powder (CB-CaP). The two powders were mixed for about 30 min in a Turbula^®^ mixer at 23 rpm in a glass bottle with zirconia balls.

### 2.2. Scaffold Fabrication

**Figure 1:**
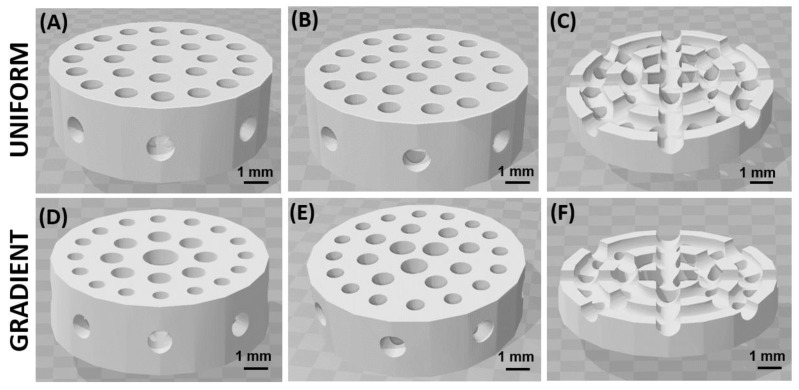
Images of the CAD models (.stl files) of the two geometries: scaffolds with pores with uniform size and pores with a size gradient. Views from both sides (**A**–**E**) and views of the cross-sections (**C**,**F**). In the cross sections: circular channels and radial channels connect the vertical pores.

The CAD models of the scaffolds were realized with the software Tinkercad^®^ from Autodesk^®^, available online. All scaffolds were designed as a disk shape with a 9 mm diameter and 3 mm thickness. Two types of scaffolds, with two different geometries for their pores, were designed and printed: (i) uniform pores of homogenous size or (ii) gradient-size pores. As shown in Figure 1, the pores are interconnected by channels in the radial direction, and in order to facilitate the cell seeding, they were designed to not pass through the whole thickness. The diameter of the uniform pores is 0.85 mm, while that of the gradient-size pores is 1.5 mm, 1 mm and 0.7 mm. These dimensions were chosen in order to have the same amount of macroporosity in the two geometries.

The scaffolds were 3D printed by P-3DP with a commercial 3D printer (ExOne Innovent+^®^, The ExOne Company, North Huntingdon, PA, USA) using an organic binder resin supplied by the producer. Briefly, the process works as follows: a moving hopper feeds the powder material on the powder bed by activating ultrasounds when it passes; then, the powder bed is flattened by two moving rollers that are fixed to the traverse, just after the hopper. A movable printhead spreads the liquid binder on selected spots, and then the binder is consolidated by the passage of a heater located just behind the hopper. The layer thickness was set to 50 µm, and the bed temperature to 80 °C. Since fine particles have a higher specific surface area and therefore require more binder to be wet, the binder saturation was increased from 100% for the pure glass-ceramic scaffolds (AP40mod) to 110% for the scaffolds with 10% of CB-CaP (10%CB-CaP). Due to the fact that the 10%CB-CaP powder showed less flowability, the powder feeding parameters were different for the two materials: recoating speed of 100 mm/s and ultrasound intensity of 50% for AP40mod; recoating speed of 20 mm/s and ultrasound of 100% for 10%CB-CaP. Moreover, the speed of the traverse and of the rollers was lower for the powder containing CB-CaP, in order to prevent fine particles from sticking to the rollers: roller traverse speed of 3 and 1.5 mm/s, roller rotation speed of 300 and 150 rpm, and rough roller rotation speed of 250 and 100 rpm for AP40mod and 10%CB-CaP, respectively.

Printing trials with 20 wt% CB-CaP were also performed, but without success. The reason is that the fine particles were sticking to the rollers even at a very low speed, with the consequent roughening of the powder bed. Moreover, the printed scaffolds were too fragile to be handled, even when the binder saturation was increased up to 150%. Pure CB-CaP scaffolds were also not possible to be printed, even when using agglomerates of CB-CaP powders produced by spray drying (Niro Atomizer Minor, Copenhagen, Denmark). In fact, although the flowability and the printing bed quality were optimal, the binder was not able to bind the agglomerates together, even with very high saturation (400%).

After printing, the scaffolds were extracted from the powder bed and dried at about 100 °C overnight. They were then cleaned from the excess powder with compressed air and sintered in a Carbolite (Derbyshire, UK) RHF-1400 furnace following these steps: heating rate 3 °C/min up to 500 °C for de-binding; heating rate 10 °C/min up to 800 °C and dwell 2 h; heating rate 10 °C/min up to 1150 °C and dwell 2 h; free cooling in the furnace. The sintering was carried out in two steps, 800 °C and 1150 °C, in order to fully crystalize the AP40mod, which undergoes two crystallizations, one starting at about 770 °C and the other at about 870 °C, as shown by TGA analyses.

### 2.3. Materials Characterization

The particle size distribution of the cuttlebone-derived powder was measured by laser diffraction using a Mastersizer 3000 from Mavern Panalytical GmbH (Kassel, Germany) following the ISO 13320 (01/2020). Approximately 0.3 g of powder was stirred into 80 mL of a 3 mol/L tetra-sodium diphosphate solution, and the resulting suspension was dispersed with an ultrasonic sonotrode (Sonopuls HD 2200, BANDELIN electronic, Berlin, Germany). The flowability of the AP40mod powder mixed with 0 to 100% CB-CaP was assessed by measuring the free settled density ($\rho_{free\;settled}$) and the tapped density ($\rho_{tapped}$), and calculating the Hausner ratio (HR) [34] as:

(1) $$HR=\frac{\rho_{tapped}}{\rho_{free\;settled}}$$

where $\rho_{tapped}$ is the tapped density of the powder after a certain number of tapping cycles. The HR is an indicator of the friction among particles, and it affects the powder flow, being powders with poor flowability characterized by a higher HR. The tapping was performed with a jolting volumeter STAV-2003 (JEL, Gablingen, Germany) using a 250 cl glass cylinder, and repeated for 1250 cycles until no change in volume was detected.

The crystallization temperatures of AP40mod and 10%CB-CaP powders were assessed by thermal analyses (TGA/DTA) using a NETZSCH Geraetebau GmbH STA 409 thermobalance (Selb, Germany). The tests were carried out by heating the samples in an alumina crucible from room temperature up to 1160 °C at 10 °C/min in flowing air. The real density of CB-CaP, AP40mod and crystalized AP40mod (heat treated with the sintering cycle) was measured with a pycnometer (Pycnomatic ATC, Porotec, Waldems, Germany).

The crystallographic phases in the materials, before and after sintering, were studied by x-ray diffraction (XRD) with a Rigaku IIID-max diffractometer, equipped with a Cu anode source (Kα radiation 1.5406 A), in the 10–80° 2-theta range, and with scan step = 0.05° and step time = 2 s. A qualitative and quantitative analysis of the XRD spectra was performed with the Rietveld-based software MAUD^©^ 2.8 [35] using the following crystallographic phases as references, which were downloaded from the COD database [36]: hydroxyapatite n. 4317043 [37], β-tricalcium phosphate n. 1517238 [38], wollastonite n. 9005777 [39], titanite n. 9000489 [40], cristobalite n. 1010944 [41] and diopside n. 1000007 [42].

The chemical elements present in the CB-CaP powder were identified by Inductively Coupled Plasma/Optical Emission Spectroscopy (ICP/OES) with a Spectro Ciros Vision CCD (125–770 nm). The CB-CaP powder was solubilized in ultrapure nitric acid (70 vol%, Sigma–Aldrich, St. Louis, MO, USA) and diluted with pure water. Calcium and phosphorus were quantified by using hydroxyapatite ultra-pure standard (>99.995% trace metal basis, Sigma–Aldrich), choosing the emission lines of 184 nm for Ca and 178 nm for P, while the analyses of the other elements should be taken as semi-quantitative. The infrared spectra of AP40mod and CB-CaP were collected with a FTIR Thermo Optics Avatar 330 over the 4000–400 cm^−1^ range in transmission mode, using KBr pellets (Houston, TX, USA).

### 2.4. Scaffold Characterization

The bulk density of the 3D printed scaffolds was evaluated by the Archimedes’ method for five specimens per each material variant, following the ISO18754:2013(E) standard and impregnating the samples in demineralized water by boiling them for 1 h. The total porosity of the 3D printed porous scaffolds was evaluated by weighing ten samples per each configuration with a laboratory scale (sensitivity 0.00001 g) and measuring their dimensions with a calliper. The total porosity was then calculated by comparing the calculated densities with the materials’ real density as measured by pycnometry. The dimensions of the pores were measured with the software ImageJ by analyzing the pictures taken with an optical microscope (Keyence VHX-100, Osaka, Japan) at 50× magnification, considering three samples per variant. The morphology of the fracture surface of 3D-printed scaffolds was investigated by FE-SEM SUPRA 40 (Carl Zeiss Microscopy GmbH, Jena, Germany).

### 2.5. Preliminary Biological Evaluations

#### 2.5.1. Cell Harvest and Cell Seeding

Human-bone-marrow-derived mesenchymal stem cell lines (hMSCs, ATCC number: PCS-500-012) were cultured in α-MEM medium supplemented with 15% Fetal Bovine Serum (FBS, EuroClone, Pero, Italy) and 1% Antibiotic/Antimycotic (AA, EuroClone), in a humidified atmosphere of 5% CO_2_ at 37 °C. The medium was changed every two days. Once they reached 70% confluence, the cells were detached by Trypsin-EDTA 1X in PBS solution (EuroClone), counted and re-suspended in standard medium with the concentration of 333,000 cells/mL.

The printed scaffolds were sterilized in autoclave at 121 °C for 15 min and then placed into 48-well plates. A total of 0.3 mL of cell suspension was added directly to each scaffold (100,000 cells/well) and to the tissue culture plate (TCP), which was used as control. The plates were incubated in a humidified atmosphere of 5% CO_2_ at 37 °C to promote cell adhesion. After a total of 24 h following the seeding, the samples with cells were moved to new plates with standard cell culture medium. Every two days until day 10, the medium was changed.

#### 2.5.2. Cytotoxicity Test (LDH Assay)

The cytotoxicity of the samples was evaluated on extracts of the 10 wt%CB-CaP bioceramic by using the lactate dehydrogenase assay LDH assay-TOX-7 (Sigma) on MRC5 cell lines (human embryonal lung fibroblast, ATCC) in vitro cultures. The samples were weighted before the extraction preparation. The extractions were prepared by soaking the samples into a standard MRC5 medium (0.2 g/mL) for 72 h at 37 °C. After 24 h of cell seeding, the medium was replaced with the extracts, and the cell culture was protracted for 24 h and 48 h to detect possible cytotoxic effects. The cells cultured in the standard medium were considered as the control group. Five replicates for each sample were considered.

#### 2.5.3. Cell Metabolic Activity Evaluation (AlamarBlue Assay)

The general metabolic activity of hMSCs on different samples was determined with the AlamarBlue Cell Viability assay (Invitrogen, Carlsbad, CL, USA) at each time point (day 3, day 5 and day 10). AlamarBlue reagent was added directly to each well at 10% of the cell culture medium volume. Then, the well plates were incubated at 37 °C in a humidified atmosphere with 5% CO_2_ for 3 h. A total of 100 μL of the solution was collected from each well. The fluorescence signal was measured with a Tecan Infinite 200 microplate reader (Tecan Group, Männedorf, Switzerland) with an excitation wavelength of 560 nm and an emission wavelength of 590 nm. TCP was used as the control group, and four replicates were considered for each experimental condition.

#### 2.5.4. Cell Proliferation Assay (DNA Quantification)

In order to evaluate cell proliferation on the different samples, a PicoGreen DNA quantification assay (Quant-iT PicoGreen dsDNA Assay, Invitrogen, Carlsbad, CL, USA) was used. Cells cultured on TCP were used as the control group. After 3, 5 and 10 days of culturing, the culture medium was removed, and the samples were washed with PBS. Samples were then covered with 300 μL of 0.05% Triton-X PBS solution and incubated at 37 °C for 1 h. Before the analysis, the samples were sonicated for 10 s with a Hielscher ultrasonic homogenizer (UP400S, 400 W-24 kHz, cycle 1, amplitude 40%, from Hielscher Ultrasonics, Teltow, Germany). A total of 100 μL of supernatant for each sample was subsequently placed in a black 96-well plate and mixed with 100 μL of PicoGreen working solution, prepared following the manufacturer’s instructions. Fluorescence intensity was measured with a Tecan Infinite 200 microplate reader (Tecan Group, Männedorf, Switzerland) using an excitation wavelength of 485 nm and an emission wavelength of 535 nm. A calibration curve was created using a double-stranded DNA standard provided by the kit and was used for the calculation of the DNA content. Finally, the approximate number of cells per sample was determined from the DNA content by the conversion factor of 7.7 pg DNA per cell. Four replicates were considered for each variant.

#### 2.5.5. Cell Distribution and Migration (Confocal Microscopy Analysis)

Cell morphology, distribution and migration were visualized by confocal microscopy after staining the samples with Oregon green phalloidin and 4′6-diamidino-2-phenylindole (DAPI). Oregon green phalloidin stains actin filaments of cytoskeleton, resulting in green fluorescence, while DAPI stains nuclei, resulting in blue fluorescence. After 3, 5 and 10 days of culturing, the samples were fixed with 4% paraformaldehyde, washed three times with PBS, and then were permeabilized using 0.2% Triton X-100 PBS solution for 30 min. After the washing by PBS for three times (15 min each time), cells were incubated in Oregon green phalloidin (5.0 μL/well) and DAPI (1.0 mL/well, 5.4 μL dilute in 25.0 mL PBS) solution for 1 h at room temperature. After three rinses with PBS, samples were observed using a Zeiss LSM 510 Meta confocal laser scanning microscope. 

#### 2.5.6. Statistical Analysis

For biological evaluations, graphPad Prism 9 (La Jolla, CA, USA) was used for statistical analysis for all the data obtained from each independent experiment. Where applicable, data were expressed as mean ± one standard deviation. The statistical analysis was performed by two-way ANOVA using the all-pair-wise multiple comparison procedure, where * *p* < 0.05 was set as the level of significance.

## 3. Results and Discussion

### 3.1. Materials Characterization

**Figure 2:**
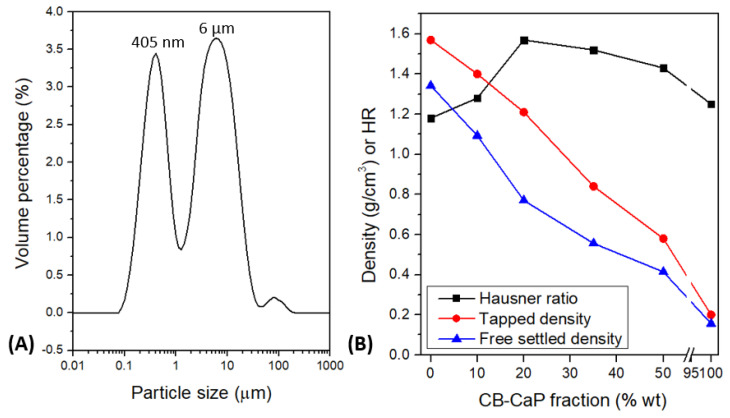
Particle size distribution of CB-CaP powder measured with laser diffraction (**A**) and tapped density, free settled density and Hausner ratio of AP40mod powder (45–100 µm) with increasing amounts of CB-CaP powder (**B**). Standard deviations for density measurements are small (<0.01 g/cm^3^) and therefore are not reported in the graph.

The particle size distribution of the CB-CaP powder shows a bi-modal distribution, with peaks at 405 nm and 6 µm (Figure 2A). Most probably, the said peaks correspond to primary particles in the range 90–1280 nm (first peak) and to agglomerates of finer particles with dimensions 1.3–45 µm (second peak), agglomerates that were not dispersed during the preparation of the sample.

As expected, the tapped density and the free settled density of the AP40mod powder (particle size 45–100 µm) decrease by adding fine particles of CB-CaP (Figure 2B). However, the Hausner ratio (HR) increases by up to 20 wt% of fine particles and then gradually decreases. This is different from what was observed by Sun et al. [33], where the HR was constantly increasing while adding fractions of 0–25 µm to the 45–100 µm powder. Even if a lower HR is usually associated with lower friction among particles and therefore with better flowability, this might not be the case. It is instead thought that the interaction forces between nanosized particles are too strong to be broken by tapping, so that the tapped density is lower than what is expected. This is confirmed by the fact that the tapped density and the free settled density of 100% CB-CaP powder are basically the same (Figure 2B), meaning that the tapping cycles did not reduce the void between particles.

**Figure 3:**
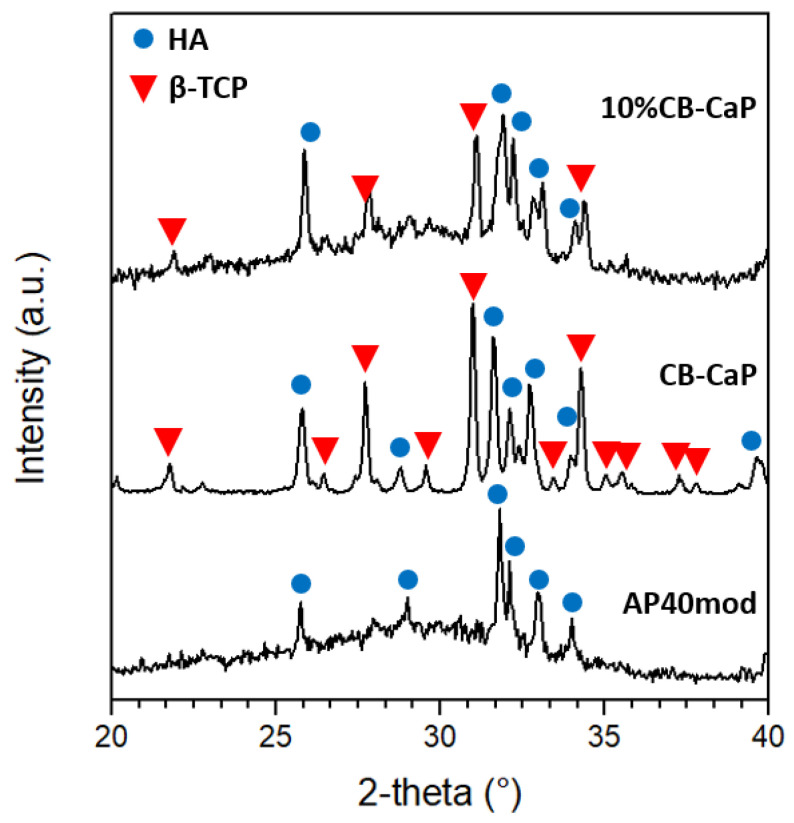
XRD spectra of AP40mod, CB-Ca-P and AP40mod/10 wt% CB-CaP mixture.

The XRD spectra of the powder materials are shown in Figure 3. The amorphous nature of the AP40mod glass is revealed by the broad, noisy band centered at about 30°. The plot also shows some small peaks corresponding to the hydroxyapatite (HA) crystalline phase. The CB-CaP powder is composed of approximately 47 wt% HA and 53 wt% β-tricalcium phosphate (β-TCP), as determined by the Rietveld analysis. This is in agreement with the ICP/OES measurements, which allowed for estimating the quantity of P and Ca in the CB-CaP powder, resulting in a Ca/P molar ratio equal to 1.58. In fact, biphasic materials of HA and β-TCP, also called BCP (biphasic calcium phosphates), have a Ca/P ratio between that of HA (1.67) and of β-TCP (1.5), depending on the relative quantities of the two phases [43]. The ICP/OES analysis also revealed the presence of small amounts of K, Na, Mg, Sr and Zn in the cuttlebone-derived powder, as a consequence of the natural origin of the material.

**Figure 4:**
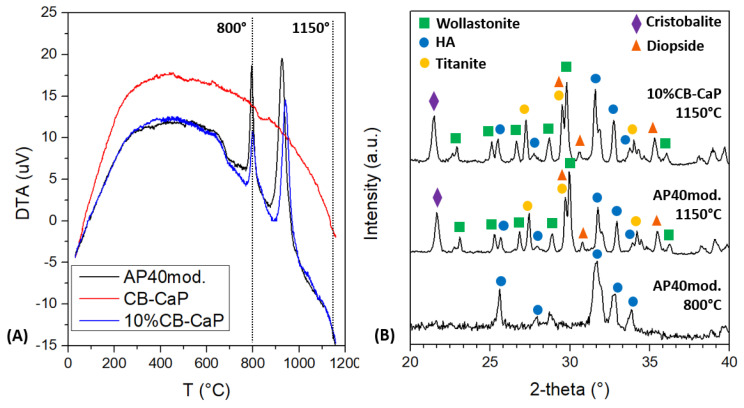
DTA analysis of the powders (**A**) and XRD spectra of the samples sintered at 1150 °C and of AP40mod treated at 800 °C (**B**).

The DTA of AP40mod shows two exothermic transformations, starting at about 770 °C and 870 °C, respectively (Figure 4A). This reveals that the glass-ceramic crystalizes in two different steps, as previously observed [33], with this being the reason for a two-step sintering process with isotherms at 800 °C and 1150 °C. Conversely, the cuttlebone-derived material does not show any exothermal or endothermal effect.

The XRD spectra in Figure 4B show that the AP40mod glass is fully crystallized after sintering and is composed of β-wollastonite—CaSiO_3_ (~34 wt%), hydroxyapatite—Ca_10_(PO_4_)_6_(OH)_2_ (~27 wt%), diopside—CaMg(SiO_3_)_2_ (~18 wt%), titanite—CaTiSiO_5_ (~16 wt%) and β-cristobalite—SiO_2_ (~5 wt%). In order to understand which phases crystalize first, some of the AP40mod powder was heat treated at 800 °C (10 °C/min, no dwell, free cooling in furnace) and analyzed by XRD. The spectrum of such a sample (Figure 4B) shows the presence of the HA phase only, while the amorphous phase broad band seems to be attenuated. One can therefore assume that the first crystallization event in the DTA plot is related to the formation of apatite, while the other phases crystalize at higher temperatures, starting at about 850–870 °C. This is in agreement with a previous work [44], where apatite was found to crystalize before wollastonite in a glass-ceramic of similar composition.

The XRD analysis also shows that the sintered sample containing 10 wt% of cuttlebone-derived powder has a phase composition very similar to the AP40mod. However, the relative quantity of HA is larger than in pure AP40mod, as pointed out by the relative intensity of the main peaks of HA and wollastonite in Figure 4B (2-theta 31.6° for HA and 29.8° for wollastonite). In fact, the quantitative analysis allowed for calculating about 36 wt% of HA and 29 wt% of wollastonite, while the relative amount of the other phases is more or less the same as in AP40mod (~16 wt% diopside; ~15 wt% titanite and ~5 wt% cristobalite). Since the β-TCP phase in the 10%CB-CaP raw powder (Figure 3) completely disappears after sintering, one can assume that β-TCP is transformed into HA, thanks to the presence of additional Ca atoms from the glass-ceramic. Therefore, the HA phase in the sintered 10%CB-CaP sample is produced from both the glass ceramic and the cuttlebone-derived material.

The real density of the AP40mod and CB-CaP powders was measured by pycnometry. The real density of CB-CaP was found to be 3.009 ± 0.016 g/cm^3^, which is just slightly lower than what was expected, it being a mixture of β-TCP (3.08 g/cm^3^ [45]) and HA (3.16 g/cm^3^ [45]). The density of AP40mod was instead found to increase with a sintering-like thermal treatment, being that the density of the crystallized powder is higher than that of the raw glassy powder (2.967 ± 0.01 g/cm^3^ and 2.875 ± 0.04 g/cm^3^, respectively).

**Figure 5:**
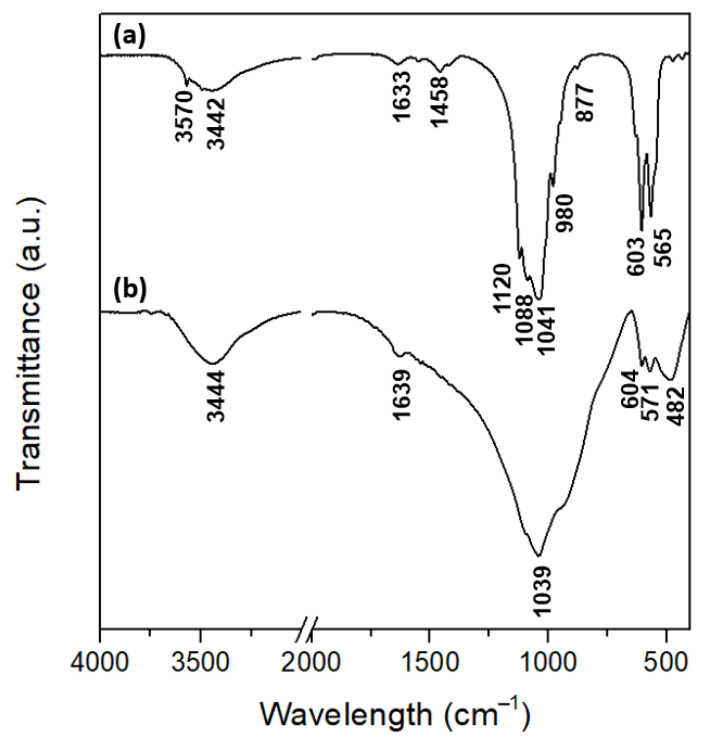
FTIR spectra of CB-CaP (**a**) and AP40mod (**b**) powders.

Figure 5 shows the FTIR spectra of the CB-CaP (Figure 5a) and AP40mod (Figure 5b) powders. The cuttlebone-derived powder shows the typical absorbance bands of HA and β-TCP, thus confirming the XRD results. The phosphate groups in HA are responsible for the peaks at 603 cm^−1^ and 565 cm^−1^ (ν_4_ PO_4_^3−^) and at 1041 cm^−1^ and 1088 cm^−1^ (ν_3_ PO_4_^3−^) [46]. The absorption bands at 980 cm^−1^ and 1120 cm^−1^ can also be attributed to PO_4_^3−^, but they are most likely related to the β-TCP phase [47]. The presence of carbonate ion substitution in HA is revealed by the small peak at 877 cm^−1^ (ν_2_ CO_3_^2−^) and by the absorption bands between 1400 and 1600 cm^−1^ (ν_3_ CO_3_^2−^) [46,48], with this meaning that the hydroxyapatite is carbonated. Moreover, structural OH^−^ groups in the hydroxyapatite lattice are responsible for the peaks at 632 cm^−1^ and 3570 cm^−1^ [46,48]. On the other hand, the infrared spectrum for AP40mod glass ceramics shows the presence of phosphate groups and Si–O bonds. The broad, strong absorption band between 800 cm^−1^ and 1200 cm^−1^ is most probably an overlap of the ν_3_ modes of PO_4_^3−^ and of the asymmetric stretching of SiO_4_^4−^ [46,49,50]. The peaks at 571 cm^−1^ and 603 cm^−1^ are also assigned to the phosphate groups (ν_4_), while the peak at 482 cm^−1^ can be assigned to the vibrations of silicate units containing non-bridging oxygens [51]. Finally, adsorbed water is present in both samples, as revealed by the absorption bands at 3442–3444 cm^−1^ [48] and at 1633–1639 cm^−1^ [52].

### 3.2. Scaffold Characterization

**Figure 6:**
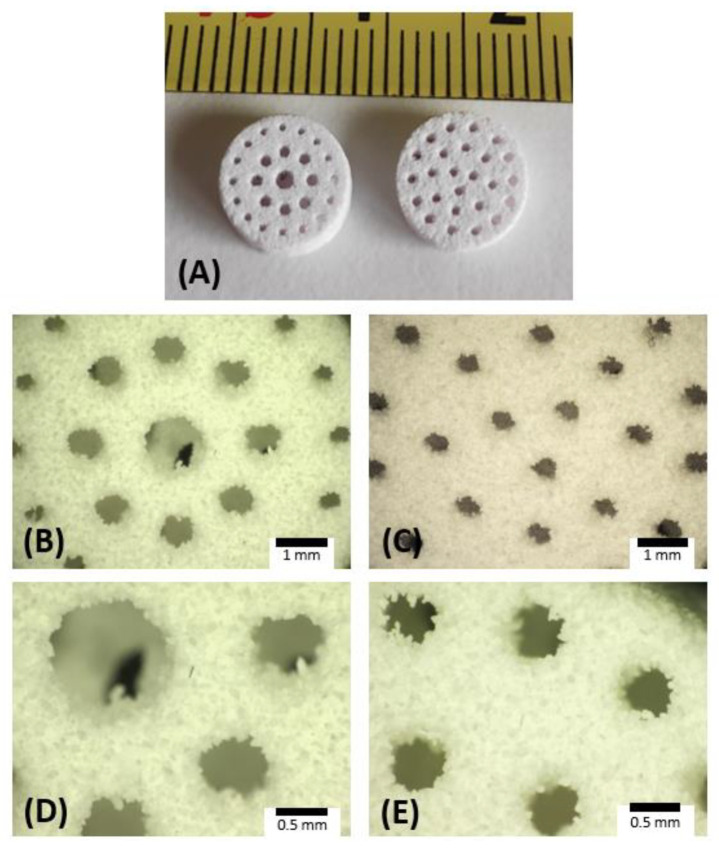
Picture of sintered scaffolds of AP40mod (**A**) and optical microscope pictures of the AP40mod scaffolds with two geometries: gradient pores (**B**,**D**) and uniform pores (**C**,**E**). Scaffolds of 10%CB-CaP look very similar to the AP40mod ones.

The 3D-printed and sintered scaffolds show a well-defined geometry with regular pore shapes, as can be seen from the pictures in Figure 6. The mass, diameter, thickness and pore dimensions of the scaffolds are listed in Table 1, together with the calculated porosity and density. The printing process showed good reproducibility, as highlighted by the relatively small standard deviations of the geometrical dimensions (<0.1 mm).

The total porosity is around ~60% and ~70% for the AP40mod and 10%CB-CaP scaffolds, respectively, thus being in the range of the typical porosity of cancellous bone [4]. The pore size in the sintered samples is smaller than in the original CAD files, obviously due to the sintering shrinkage. For example, the size of uniform pores was designed to be equal to 0.85 mm, while it is 0.47 mm and 0.50 mm in the sintered AP40mod and 10%CB-CaP scaffolds, respectively. Shrinkage occurred mostly in the radial direction, as the final diameters are 8.4–8.5 mm (9 mm in the CAD), while the thickness remains more or less unchanged, around 3 mm. Moreover, shrinkage is greater in the case of the pure AP40mod samples, which have less porosity and therefore show higher densification during sintering. This is confirmed by bulk density measurements, equal to 1.57 g/cm^3^ and 1.40 g/cm^3^ for the AP40mod and 10%CB-CaP scaffolds, respectively. Accordingly, the apparent porosity is equal to ~47% (AP40mod) and ~53% (10%CB-CaP).

**Figure 7:**
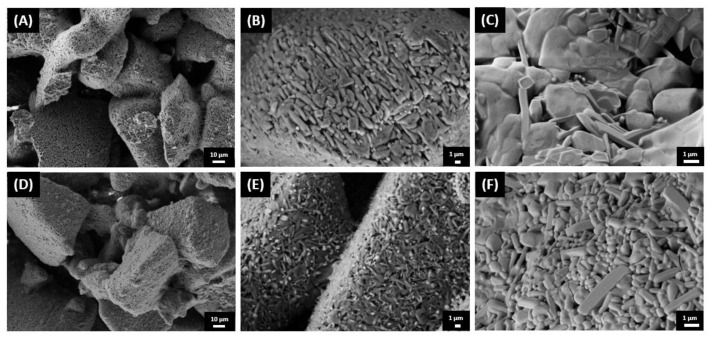
FE-SEM pictures of the scaffolds’ fracture surface: AP40mod (**A**–**C**) and 10%CB-CaP (**D**–**F**).

The higher densification achieved by pure AP40mod scaffolds can be explained by taking into account the different properties and particles size distributions of the two used raw materials. AP40mod has an amorphous structure and is therefore more prone to densify by viscous flow sintering. However, in the 10%CB-CaP scaffolds, the cuttlebone-derived nanoparticles very likely surround the bigger glass-ceramic grains, acting like a shield for the glassy phase. Indeed, the FE-SEM pictures (Figure 7) show that the surface of the AP40mod big particles (45–100 µm) are covered by a considerably large number of very fine particles, with dimensions < 1 µm. The image at its highest magnification (Figure 7C) shows that AP40mod particles are formed by grains of different sizes and shapes, most probably corresponding to the different crystallographic phases detected by XRD. On the other hand, the picture of 10%CB-CaP scaffold (Figure 7F) presents a much finer texture, which is formed by smaller HA crystals. At lower magnifications, the presence of small particles covering the bigger ones can be intuitively noticed by the smooth, silky texture of the surfaces in Figure 7D, differently from the pure AP40mod in Figure 7A.

### 3.3. Preliminary Biological In Vitro Tests: Cytotoxicity and Cytocompatibility

The cytotoxicity test was performed by the LDH assay on the extraction of the 10%CB-CaP bioceramic with different geometries. The cell death ratio was almost the same as for the negative control, suggesting there was no cytotoxicity induced by the material or by the printing process.

**Figure 8:**
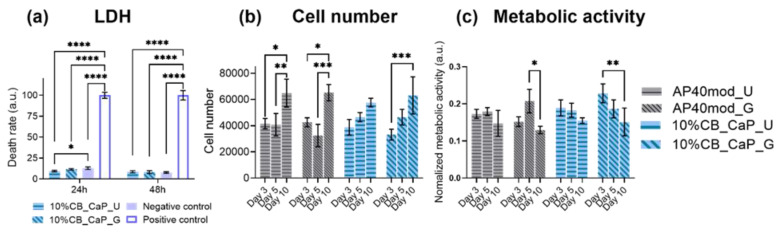
Cytotoxcity (**a**) of the scaffold of 10%CB-CaP at 24 h and 48 h, cell proliferation (**b**) and metabolic activity (**c**) of hMSCs on day 3, 5 and 10 of cell culturing. N = 8; * *p* < 0.05; ** *p* < 0.01; *** *p* < 0.001; **** p < 0.0001. U: uniform pores; G: gradient pores.

Cell proliferation and metabolic activity of hMSCs on different scaffolds were measured on day 3, 5 and 10 of cell culturing in a standard culture medium. The results are shown in Figure 8. A gradual increase in the cell numbers is observed in all groups during the 10 days of culturing (Figure 8a), although this is not significant in the first five days. There are slight differences in the metabolic activity of different materials and geometries. The AP40mod samples show a peak on day 5, especially for the material printed with gradient pores, while the metabolic activity shows a gradual decrease over time in the presence of the cuttlebone-derived particles; in particular, the scaffolds with gradient pores show a significant drop in metabolic activity from day 3 to day 10.

**Figure 9:**
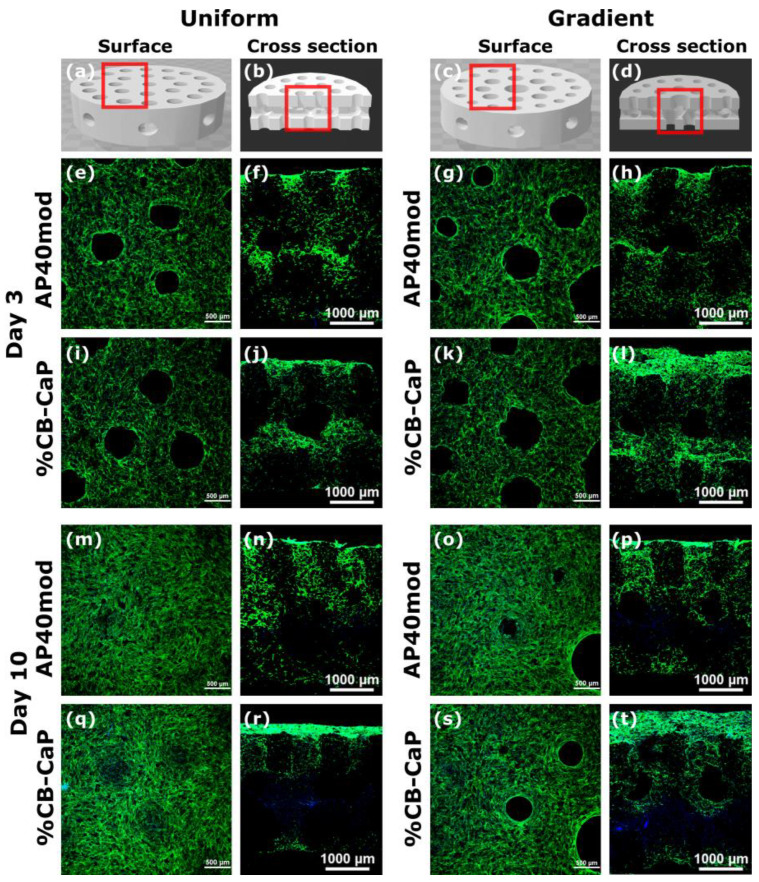
Confocal images of the cells seeded on the scaffolds and cultured for 3 and 10 days. The cytoskeleton is highlighted in green and the cells’ nuclei in blue. AP: AP40mod (**e**,**f**,**g**,**h**,**m**,**n**,**o**,**p**); CB: 10%CB-CaP (**i**,**j**,**k**,**l**,**q**,**r**,**s**,**t**). Images of the surface (**a**,**c**) or of the cross-section (**b**,**d**).

The cell distribution (surface) and migration (cross section) on the bioceramic scaffolds are shown in Figure 9. The images are consistent with the results of cell proliferation. In general, all samples promote fast cell adhesion at day 3, and they are able to create a monolayer on the scaffold surface to cover the small pores at day 10. Moreover, the adhered cells of all groups penetrate in the pores colonizing the 3D structure, as shown in the cross-section images. There are no significant differences in the cell behavior among the different groups.

It was already proven in previous research [31] that the presence of cuttlebone-derived powder can provide strontium, useful to enhance the scaffolds’ bioactivity. In the current study, there are no significant differences in terms of cell numbers up to 10 days among different groups, probably due to the good bioactivity of AP40mod and to the relatively low amount of cuttlebone-derived powder (10 wt%). Therefore, cell proliferation does not appear to be affected by the presence of CB-CaP or by the 3D-printed architecture. Differently from cell proliferation, the metabolic activity of hMSCs is instead affected by the pore geometry, with the scaffolds with gradient pores showing a decrease in their metabolic activity at day 10. According to the literature [53], by regulating the energy production and the generation of substrates for biosynthetic pathways, the metabolic pathway could regulate the differentiation of stem cells. This suggested that the decrease in metabolic activity of hMSCs observed on day 10 could be due to the differentiation of hMSCs [54].

## 4. Conclusions

Bone scaffolds with two different geometries—uniform or gradient pore sizes—were fabricated by binder jetting 3D printing in order to investigate the effect of the geometry of the pores on the cellular behavior. In addition, nanosized biphasic calcium phosphate (BCP) powder derived from cuttlefish bones was used, mixing it with a glass-ceramic powder (AP40mod) with a particle size of 45–100 µm. The maximum amount of nanopowder that was possible to add while obtaining a good printing quality was 10 wt%. The sintered scaffolds were found to be composed mainly by hydroxyapatite and wollastonite, while diopside, titanite and cristobalite were present in smaller amounts. The amount of HA was higher when the nanopowder was added, since BCP converts into HA upon sintering. The scaffolds’ porosity was found to increase from 60% to 70% with the addition of the nanosized powder, which very likely acts as a barrier for the glass-ceramic densification by covering the surface of the big glassy particles and limiting their mutual contact. No significant differences in terms of cell proliferation were found among samples with different pore geometries and with or without the nanopowder. However, while the cell number increases over 10 days of culturing, the metabolic activity shows a gradual decrease over time, especially for scaffolds with gradient pores and cuttlebone-derived powder. This might be associated with cell differentiation, but additional tests are needed to clarify this point. In general, the biological in vitro tests show that all scaffolds promote the fast adhesion of human mesenchymal stem cells (hMSCs), which also penetrate the pores and colonize the porous structure.

## Figures and Tables

**Table 1:** Dimensions, mass, density and porosity of the scaffolds.

Material	CAD Model		AP40mod		10%CB-CaP	
Pores Geometry	Grad.	Unif.	Gradient	Uniform	Gradient	Uniform
Diameter (mm)	9.0	9.0	8.41 ± 0.05	8.45 ± 0.08	8.46 ± 0.09	8.48 ± 0.09
Thickness (mm)	3.0	3.0	3.11 ± 0.06	3.09 ± 0.06	2.96 ± 0.07	2.94 ± 0.89
Mass (mg)			197 ± 2	195 ± 6	150 ± 5	148 ± 6
Pores size (mm)	1.5 1.0 0.7	0.85	1.17 ± 0.03 0.62 ± 0.05 0.34 ± 0.05	0.47 ± 0.04	1.26 ± 0.03 0.74 ± 0.07 0.44 ± 0.07	0.50 ± 0.06
Total porosity ^a^ (%)			61	62	70	70
Bulk density ^b^ (g/cm^3^)			1.57 ± 0.02		1.40 ± 0.09	
Apparent porosity ^b^ (%)			47 ± 4		53 ± 3	

^a^ Calculated by dividing the mass by the volume and dividing by the theoretical density measured by pycnometry. ^b^ From Archimedes’ tests.

## Data Availability

The data presented in this study are available on request from the corresponding author.
